# Serum and saliva protein levels in females with breast cancer

**DOI:** 10.3892/ol.2014.2535

**Published:** 2014-09-15

**Authors:** SABAH ISA AL-MUHTASEB

**Affiliations:** Department of Medical Allied Science, Zarka University College, Al-Balqa Applied University, Amman 11196, Jordan

**Keywords:** breast cancer, saliva proteins, serum proteins

## Abstract

The aim of the present study was to investigate the change in the total protein content between the serum and saliva of female patients with breast cancer and in healthy females. The study was conducted between October 2012 and November 2013. There were 80 females in the present study with 40 breast cancer patients and 40 healthy control subjects, with an age range of 50–70 years. The results of the study showed that the mean value ± standard deviation of the total serum protein in patients with breast cancer was 7.63±0.41 g/dl, whereas in the healthy subjects it was 6.14±1.84 g/dl. The total salivary protein measurement was 0.14±0.07 g/dl and 0.25±0.09 g/dl in the breast cancer and healthy group, respectively. Therefore, it can be concluded that the total serum protein was higher in female patients with breast cancer, whereas the levels in the saliva were lower compared to the healthy female group. The results of the present study indicate that serum protein levels may be used for the diagnosis of breast cancer.

## Introduction

Proteins play a central role in cell structure and function. The serum contains a mixture of proteins that differ in origin and function, and the amount of protein in the vascular compartment depends on the balance between the rate of synthesis and the rate of catabolism or loss (1). It is a well-established and evidence-based fact that serum protein levels may undergo changes during the process of breast cancer (2). Proteins are also present in other tissue fluids, in addition to serum (1).

Breast cancer presents a major healthcare burden to females as it is the most common type of cancer occurring in females worldwide (3) and is the most common type of cancer in Jordan. Breast cancer is responsible for 20% of the total cancer cases with a 22% mortality. The age-standardized incidence rate of breast cancer increased from 29/100,000 in 1996 to 50/100,000 in 2008, and therefore, early diagnosis is of great importance in reducing mortality (4). However, the biological and clinical progression of breast cancer is not easily predicted, as there are a number of types of this disease that behave differently between patients (5). It is well known that proteins are the elemental constituents of all living cells and they are included in numerous substances, including enzymes, hormones and antibodies. Changes in the concentrations of serum protein have been associated with cancer disease processes and can be indicative of health problems that may provide important diagnostic information (1). The amount of protein in the serum depends on the balance between the rate of its synthesis, and that of its catabolism or loss. A total plasma protein test measures the total amount of protein in the plasma, as well as the amounts of albumin, globulin and fibrinogen (6), and it also shows the actual functioning of an organism (7).

Saliva is an important biological fluid for the detection of physiological and pathological conditions of the human body (8,9). Whole saliva is a mixed fluid that is predominantly derived from three pairs of major salivary glands, submandibular (70%), parotid (25%) and sublingual (5%) (10), and the remainder is from the minor salivary glands that are located at various oral mucosal sites. Increasing attention has focused on diagnosis by saliva-based analysis of biologically active compounds, as saliva has been shown to represent the clinically relevant compounds (11,12). Saliva analysis is able to prevent, predict and diagnose numerous health problems and diseases (13), and the methodology for saliva collection is a simple, non-invasive method. Human saliva contains clinically relevant proteins and ~30% of blood proteins are also present in the saliva, emphasizing its use in clinical applications. Previously, attention has bene given to salivary analysis, not only for detecting abundant proteins, but also for the detection of other components, including pollutants, hormones, enzymes, amino acid, proteins, statherin, histatin, mucin and cystatins (14), and their association with bacterial and viral infections, as well as being an indicator for systemic diseases (15–17). The major protein species in the saliva have extensively undergone post-translational modifications, including phosphorylation, sulfation and glycosylation (18,19). The aim of the present study was to determine the total protein levels in the saliva and serum from female patients with breast cancer in comparison to the levels in the healthy control group.

## Materials and methods

### Subjects

The present study was conducted at different public hospitals in Amman (Jordan) and at the Medical Allied Sciences Department, Zarka University College (Amman, Jordan) between October 2012 and November 2013. The study included two groups of females, aged 50–70 years, and an evaluation of a full medical history was completed for any pre-existing systemic diseases. The first group consisted of 40 female patients, diagnosed with breast cancer by clinical and histological analysis, and the second group was 40 healthy females subjects that constituted the control group. The total protein concentrations obtained from these two groups were measured in each saliva and serum sample.

### Specimen collection

Venous blood samples were obtained from all patients in plain tubes, and following coagulation the sera were separated and stored at −20°C until analysis. Simultaneously, the saliva was also collected.

The collection of the saliva involved obtaining 2 ml of unstimulated whole saliva under a resting condition ≥1 h after eating and drinking. The patients and control subjects were asked to wash their mouths several times with de-ionized water. Subsequently, the saliva that was accumulated in the floor of the mouth, under the tongue, was drawn by plastic pipettes (20). The saliva samples were centrifuged at 699 × g for 10 min to eliminate any debris. The samples were frozen immediately and stored at −20°C until analysis.

### Estimation of total protein in the saliva and serum

The salivary and serum total protein estimations were conducted using the Biuret method (21). The method for the total protein is founded on the method proposed by the American Association for Clinical Chemistry and National Committee for Clinical Laboratory Standards (22). The principle of the method depends on the enzymatic reaction sequence used in the assay of the total protein. The total protein was determined using a Roche Cobas automated clinical chemistry analyzer (Roche Diagnostics GmbH, Mannheim, Germany Systems).

### Statistical analysis

The collected data were analyzed by Excel (2010), using the Statistical Package for Social Sciences (SPSS Ver. 19; SPSS, Inc., Chicago, IL, USA) for the inferential statistics. For the purpose of data presentation and interpretation, the patient cases were presented as figures to indicate the changes in total protein in healthy individuals compared to breast cancer patients. The t-test was used to analyze significant differences between healthy individuals and breast cancer patients. P<0.05 was considered to indicate a statistically significant difference. Data are presented as mean ± standard deviation.

## Results

### Total proteins in the serum and saliva

**Figure 1:**
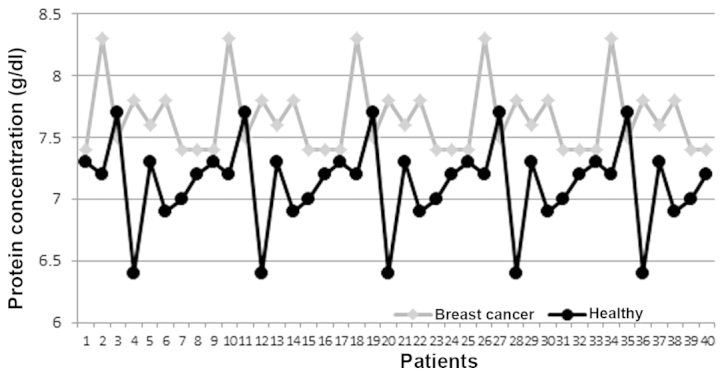
Concentrations of total serum protein in female patients with breast cancer and in the healthy group.

**Figure 2:**
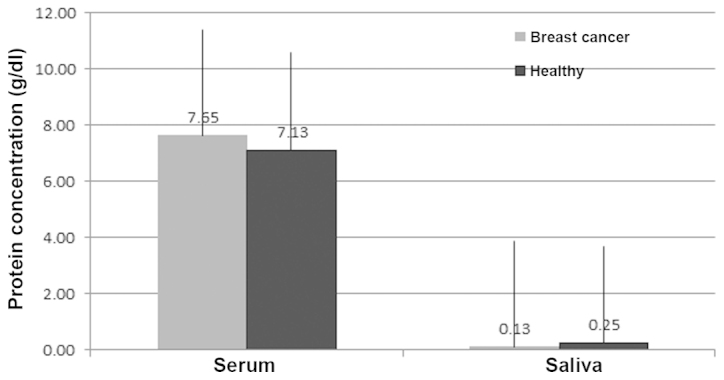
Mean concentrations of proteins in the serum and saliva of patients with breast cancer and the healthy group.

**Figure 3:**
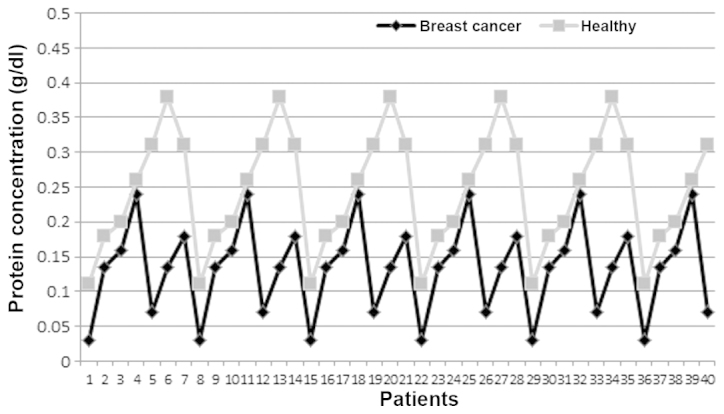
Concentrations of total saliva protein in female patients with breast cancer and in the healthy group.

Table I shows the total protein concentrations in the serum and saliva of female patients with breast cancer and the control group. The results show that the levels in the serum of the control group compared to those levels in breast cancer patients were 6.14±1.84 and 7.63±SD 0.41 g/dl, respectively (Figs. 1 and 2). The mean values of the protein concentration in the serum of breast cancer patients were significantly higher than those in healthy individuals with a statistical significance (P<0.05), whereas the mean levels of the saliva protein concentrations in the breast cancer group were lower than the mean level for the healthy individuals, with 0.25±0.09 and 0.14±0.07 g/dl in the healthy controls and the patients with breast cancer, respectively (Figs. 2 and 3). The differences were statistically significant (P<0.05).

## Discussion

Breast cancer is estimated to be the most commonly diagnosed neoplasm in females according to the latest statistics from the Jordan National Cancer Registry (23). In 2008, 864 females were diagnosed with breast cancer, which constitutes 18.8% of the overall new cancer cases. Breast cancer came first among the types of cancer in females, accounting for 36.7% of total female cancers, and it has been shown to be the foremost cause of cancer mortalities among females in Jordan. The median age at diagnosis is ≥51 years (24).

In the present study, the measurements of the serum total protein revealed that there was an increase in the total protein concentration in the serum of female patients with breast cancer. This result is in accordance with several previous studies that have explored the evaluation of serum total protein in patients with a brain tumor (1), and it also concurs with a study that found significant increases in several protein profile levels in the sera of lung cancer patients, using protein microarray and immunoassay techniques (25). This increase in total serum protein concentration can be due to the fact that total serum protein is composed of albumin and other proteins, collectively termed as globulins, and it is known that the serum albumin concentration may change under oxidative stress, such as the stress associated with cancer (26). In addition, as the plasma circulates through the tissues, it collects proteins that are released from their original locations due to certain physiological events, including tissue remodeling, trauma and cell death, which lead to an increase in total serum protein. The ability of differentiating between the proteins that truly reside in the plasma with those that are present following their release due to physiological events, remains to be resolved. However, the latter is inconsistently found, and is usually only present at an extremely low concentration. By contrast, the present results differ with the results of a study conducted by Jsiem *et al* (27) in the evaluation of serum and/or tissue proteins in patients with bladder cancer and in patients with gynecological malignancies (28). However, with regards to the levels of the total saliva protein of patients with breast cancer, the present study revealed a decrease in the total saliva protein compared to the control group. This result is in accordance with the results from the study by Ozturk *et al* (29) who assessed female patients with breast cancer who underwent chemotherapy treatment.

The original sources of the total proteins present in the saliva may be affected by the patient conditions and type of treatment. Among these sources are leakage of plasma into the saliva and the outflow of gingival crevicular fluid (30). There are two known transport methods for the molecules that are not part of the normal salivary secretions from the serum to the saliva. These are the transcellular route by passive diffusion and active transport, and the paracellular route (ultrafiltration) through tight junctions (31). However, the exact mechanism of plasma-protein leakage and whether there is selective transport of specific plasma proteins into the salivary system, remains unknown.

Another reason for the decrease of the total protein in the saliva may be due to the transport system in which the transport depends on the polarity and the charge of the molecule. Thus far, the majority of the identified biomarkers are not part of the intrinsic components of saliva, but are small molecular-weight inflammatory markers derived from the serum that are transported into the saliva. Additionally, the decrease in the total protein in the saliva may be due to the quantity and constitution of secreted human saliva, which depends on particular factors, including flow rate, circadian rhythm, type and size of the salivary gland, duration and type of stimulus, diet, drugs, age, gender, blood type and physiological status (8).

Saliva is a ‘real-time’ fluid due to the exocrine salivary glands that generate protein profiles, which are representative of an individual’s health and well-being status at the time of collection (32). It is known that blood is contained within a closed-loop circuit, whereas the saliva is continually produced and excreted in an open-ended circuit. Thus, as a circulating media, saliva may be a more useful biological fluid than blood for representing the protein profile as it is easier to test than blood, and is altered in the presence of malignant diseases (33). As the subjects in the present study were exposed to chemotherapy treatment, this may be the decisive factor for the decrease in the total protein in the saliva. The changes in the salivary protein can now be observed quantitatively in different physiological or pathological stages (34). However, in order for a valid analysis, there are necessary precautions that can be followed to avoid proteolysis, deglycosylation and dephosphorylation of salivary proteins by microbial and host enzymes in the saliva. In the present study, precautionary measures were considered. Breast cancer is one of the conditions that initiate the acute phase response by increasing the levels of specific hepatic proteins, such as positive acute-phase proteins (35). At the same time, there are groups of proteins in the body that have been reported to decrease in concentration due to the enhancement in their catabolism, rather than in their synthesis. These proteins are known as negative-phase proteins, including albumin and prealbumin (36). Therefore, the detection of hypoproteinemia can be the result of the net balance between the protein synthesis of positive acute-phase proteins, and the catabolism of negative acute-phase proteins. Protein modifications, including glycosylation, phosphorylation and proteolysis, occur in a dynamic environment that is determined by the continual supply of newly synthesized proteins and the removal of proteins by swallowing. Evaluating the whole saliva proteome in a continuous turnover environment is necessary for understanding the physiological and pathological processes that are relevant to oral health, and may be critical for the identification of important biomarkers (19).

## Figures and Tables

**Table I:** Total protein in the serum and saliva from females with breast cancer and the control group.

Group		Mean±SD	P<0.05
TP serum	Normal	6.14±1.84	0.013
	Breast cancer	7.63±0.41	
TP saliva	Normal	0.25±0.09	0.043
	Breast cancer	0.14±0.07	

SD, standard deviation; TP, total protein.
